# Protein sector analysis for the clustering of disease-associated mutations

**DOI:** 10.1186/1471-2164-15-S11-S4

**Published:** 2014-12-16

**Authors:** Jose Guevara-Coto, Charles E Schwartz, Liangjiang Wang

**Affiliations:** 1 Department of Genetics and Biochemistry, Clemson University, Clemson, SC 29634, USA; 2 J.C. Self Research Institute of Human Genetics, Greenwood Genetic Center, Greenwood, SC 29646, USA

**Keywords:** Co-evolving residues, statistical coupling analysis, protein sectors, disease phenotype

## Abstract

**Background:**

The importance of mutations in disease phenotype has been studied, with information available in databases such as OMIM. However, it remains a research challenge for the possibility of clustering amino acid residues based on an underlying interaction, such as co-evolution, to understand how mutations in these related sites can lead to different disease phenotypes.

**Results:**

This paper presents an integrative approach to identify groups of co-evolving residues, known as protein sectors. By studying a protein family using multiple sequence alignments and statistical coupling analysis, we attempted to determine if it is possible that these groups of residues could be related to disease phenotypes. After the protein sectors were identified, disease-associated residues within these groups of amino acids were mapped to a structure representing the protein family. In this study, we used the proposed pipeline to analyze two test cases of spermine synthase and Rab GDP dissociation inhibitor.

**Conclusions:**

The results suggest that there is a possible link between certain groups of co-evolving residues and different disease phenotypes. The pipeline described in this work could also be used to study other protein families associated with human diseases.

## Background

The role of mutations in disease phenotypes is an important focus for human genetics [1-3]. The identification of mutations and their link to a disease has generated numerous data entries which should optimally be accessible for ongoing research efforts. This has resulted in the establishment of databases such as the Online Mendelian Inheritance in Man (OMIM) [4], where it is possible to access data regarding a gene or gene product and browse through the information of how different mutations are associated with reported phenotypes. Even though this represents a valuable resource for clinical and molecular studies, the challenge of determining if differences in the phenotypes (severity, expansion of symptoms) within a syndrome can be associated with changes in specific groups of residues remains to be fully resolved. However, recent approaches in the study of proteins and their evolution have opened a door to analyze proteins from a different view [5-9] and attempt to associate reported clinical phenotypes to groups of correlated residues.

The techniques such as statistical coupling analysis (SCA) and direct coupling analysis (DCA) amongst others [5-7,10] have approached the study of proteins by focusing on the idea of residue co-evolution within the protein super-family [6,8,11]. These residues, whose interaction is not hindered by their spatial distribution, are organized within groups that have a seemingly underlying evolutionary relationship amongst them. Such groups of residues have been termed protein sectors and have been identified in protein super-families that comprise various lineages [6,10]. These protein sectors have been characterized as important to the protein by either contributing to its biological identity or its function [5,9].

The statistical coupling analysis requires the use of a large number of sequences as well as structural models for a representative member of the protein family to be analyzed [6,7]. Although some protein structures are still not available, the advancement in crystallography accessibility as well as sequencing technologies coupled with increasing computational power and protein modeling accuracy and efficiency, has made it possible to obtain large sets of data in order to explore these approaches in multiple protein families, including those of clinical significance in humans [12-14].

Previous studies have focused on identifying co-evolving residues, and therefore understanding how these correlated amino acid units, and the mutations within these regions, could affect the protein. The most common approaches consist of biochemical assays that provide experimental support to the link between correlated residue units and measureable characteristics such as stability or catalytic activity [6,11]. However, it is important to note that most of these studies have focused primarily on the analysis of regions with an associated function, such as domains, thus concentrating only on a segment of the totality of residues that comprise the proteins.

Although the study of specific regions provides valuable information to further understand how changes in amino acids can affect the overall function of the protein, the exclusion of residues outside the defined region could however lead to a possible loss of valuable information regarding residue distribution in functional sectors. This is of special importance in human genetic disorders, where diseases have multiple phenotypes ranging in severity, which can be associated with the location of a mutation within the protein.

We propose an integrative approach that consists of analyzing full-length sequence alignments from proteins and the subsequent identification of protein sectors using statistical coupling analysis [6]. The identified sectors and known mutation data from OMIM as well as other information resources were then used to determine a possible link between the location of an amino acid residue change and disease phenotype. Our results based on test case proteins known to be associated with X-linked intellectual disabilities revealed that it might be possible to associate disease variants to protein sectors.

## Methods

**Figure 1:**
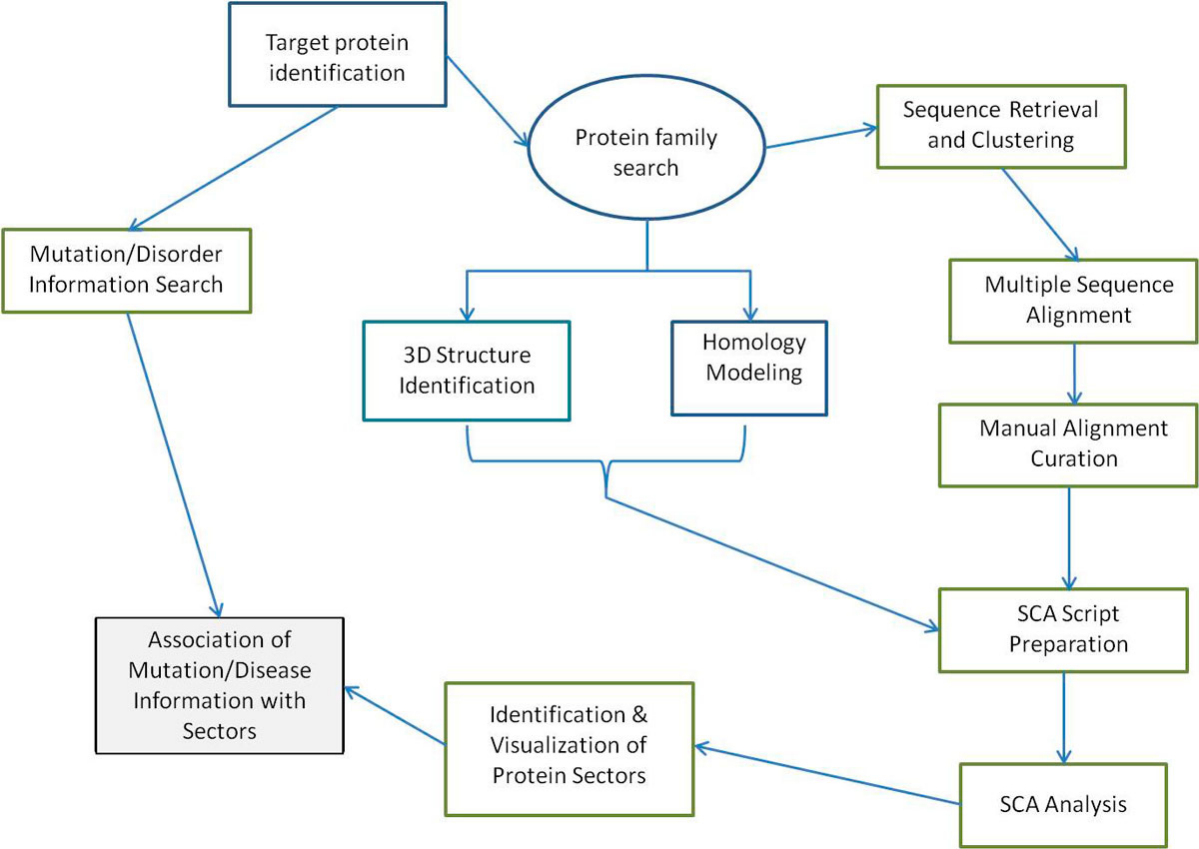
**Proposed pipeline for analyzing co-evolving residues from protein families associated with human diseases**. The flow chart includes the alternative of using homology modeling or other protein modeling methods if the 3D structure is not available. The association of disease information with protein sectors is in a grey shaded box because it is the end result of the analysis.

As shown in Figure 1, the pipeline used in our work consisted of three different stages: (1) dataset acquisition, multiple sequence alignment and curation; (2) identification of protein sectors and mapping of disease-causing mutations; and (3) association between protein sectors and disease phenotypes.

### Dataset acquisition

The first step was to obtain a reference protein sequence. Selection of the sequence and its organism is determined based on the type of dataset to be analyzed. The retrieval of homologous sequences belonging to the protein family to be analyzed was done using one iteration of PSI-BLAST with a maximum number of target sequences set to 1000. This allowed us to obtain multiple sequences representing diverse evolutionary lineages. The motivation behind this was to enrich sequence diversity, thus allowing for the possible identification of groups of conserved co-evolving residues. Another reason to attempt to identify various sequences was to diminish the possible bias towards an organism that has overrepresentation of entries in the database. The approach of protein sector analysis may not be applied to disease genes with few homologous sequences in the database. However, this is not supposed to be a common situation with more and more genomes being sequenced.

BlastClust [15] was subsequently used to reduce sequence redundancy within the dataset. The parameters used were: similarity threshold of 85% and 100% coverage. This helped diminish the over-representation of highly similar proteins with different entry numbers. Clustering of sequences further reduces the number of partial sequences that could cause alignment discordances due to their limited size. Further manual curation of the sequences was done in order to generate a high-quality sequence dataset for the alignment. In our pipeline, we implemented conditions to maintain dataset quality which primarily consisted of elimination of low quality predicted proteins and discarding of partial sequences.

### Protein structure retrieval

Protein structure files for the reference sequences used in the test cases were obtained from the Protein Data Bank [16]. The entry numbers were obtained by running a BLAST search with the reference sequence and setting the PDB as the search database. Highest scoring matches were then selected and downloaded from the PDB website. The PDB file was used to map the multiple sequence alignment to the residue positions defined in the structure file. This information was required for analyzing each site to identify residue co-evolution.

An alternative for proteins without a 3D structure is the use of homology modeling. We generated such homology structure files for our test sets by using the freely available software such as CPHmodels 3.2 [17] or the Swiss-Model server [18]. The resulting PDB files could be used in the pipeline. This alternative requires the use of a separate numbering file for the residues, which can be included in the pipeline.

### Multiple sequence alignment

Each of the working datasets was subsequently aligned using ClustalW [19] incorporated in MEGA5 [20]. The parameters were set as default for gap penalties and the substitution matrix selected was BLOSUM62 [21]. After alignment, manual adjustments were undertaken to further curate the alignment file. These modifications included the elimination of high gap-creating sequences and trimming of large sequences (based on comparison of starting and ending residues of the reference or "seed" sequence). After this was accomplished, gaps were eliminated and sequences were realigned using the same algorithm and parameters. The final alignment files varied in the number of sequences, but for each test case there were more than 250 amino acid sequences in each file.

### Protein sector identification

The statistical coupling analysis was undertaken to identify the groups of coevolving residues. The SCA toolbox for MATLAB was obtained from the Rama Ranganathan laboratory at the Green Center for Systems Biology, UT Southwestern Medical Center. The MATLAB script was modified and adapted to analyze the test cases.

The identification of protein sectors was accomplished using the methodology previously described [6,11]. This consists of the calculation of the degree of conservation for each amino acid position. Subsequently, the statistical coupling analysis generates a positional correlation and a sequence correlation matrix. This was followed by a spectral decomposition and analysis of the statistically significant eigenvalues, and an independent component analysis, which further defines the identified protein sectors. Finally, the protein sectors are mapped onto the primary structure of the protein based on their position in the structure file. This information can also be used to generate a 3D structure with different protein sectors (For further information refer to Halabi et al [6]).

### Mapping of mutations within protein sectors

The information associated with mutations in a protein family can be obtained from databases such as OMIM as well as publications related to the protein family, their clinical importance and reported phenotypes known to be associated with the mutations. The clustering or grouping of mutations can be based on their location, within one of the major regions: N-terminal, central/other, and C-terminal. This allows for the preliminary classification of the mutations as well as the latter characterization of the residues identified within the protein sectors.

The labeling and numbering of residues and their corresponding sectors was done using a PDB file obtained by BLAST search using the reference sequence for each family as a query. The full-length sequence was used to maximize the number of possible residues belonging to protein sectors. By increasing the length and coverage of the alignment, it is possible to identify co-evolving residues outside the main protein domain, which could be associated with a disease phenotype.

### Visualization of sectors in the protein 3D structure

The program Jmol http://jmol.sourceforge.net/ was used to visualize the 3D structures in PDB files. Protein domains were subsequently identified and colored using the information available from PDB. Each domain was assigned an arbitrary color, based on visualization purposes (not functional association). For the visualization of protein sectors, the residues identified were assigned the color that corresponded to the group of residues to which they belonged based on the SCA analysis.

## Results and discussion

### Identification of protein sectors

The selection of spermine synthase (SMS) and the Rab GDP dissociation inhibitor 1 (GDI1) as test cases was based on the available information for both proteins. SMS has been widely studied, with multiple mutations and the associated phenotypes comprehensively described as well as a vast amount of information on the importance of this molecule in brain development [22-25]. These characteristics as well as the fully resolved crystal structure (PDB: 3C6K) made SMS a good candidate as a test case.

GDI1 has been associated with non-specific X-linked intellectual disability, with different mutations being reported [26,27]. This represents an interesting study case to determine if co-evolving residues could possibly be associated with disorders that appear to have clinical and genetic heterogeneity [26]. This protein also has an available crystal structure (PDB: 1LV0), which made it a suitable test case to analyze with the proposed approach.

The test cases of SMS and GDI1 were analyzed using full-length sequences and their corresponding structures. The results reported in this paper were obtained using a personal computer (Intel Core i7 Q720 at 1.60 GHz, 4.00 GB RAM) installed with the MATLAB 2012a and SCA packages. The analysis covered 90% of the residues represented in the crystal structures of SMS [28] and GDI1 [29]. This allowed for further identification of sector-grouped residues, located across the covered area of the protein. A possible issue is the need of protein structural data for the analysis. Such data may not be always available. However, this can be addressed using methods such as homology or *ab initio *modeling. Since the analysis of residue co-evolution is based on the multiple sequence alignment, the accuracy of homology modeling does not represent an issue to the approach. The structure model is only used to map the positions of the multiple sequence alignment to the primary structure of the protein. Thus, the modeled structure file can be used in the pipeline in the same manner as the files obtained from the Protein Data Bank.

**Figure 2:**
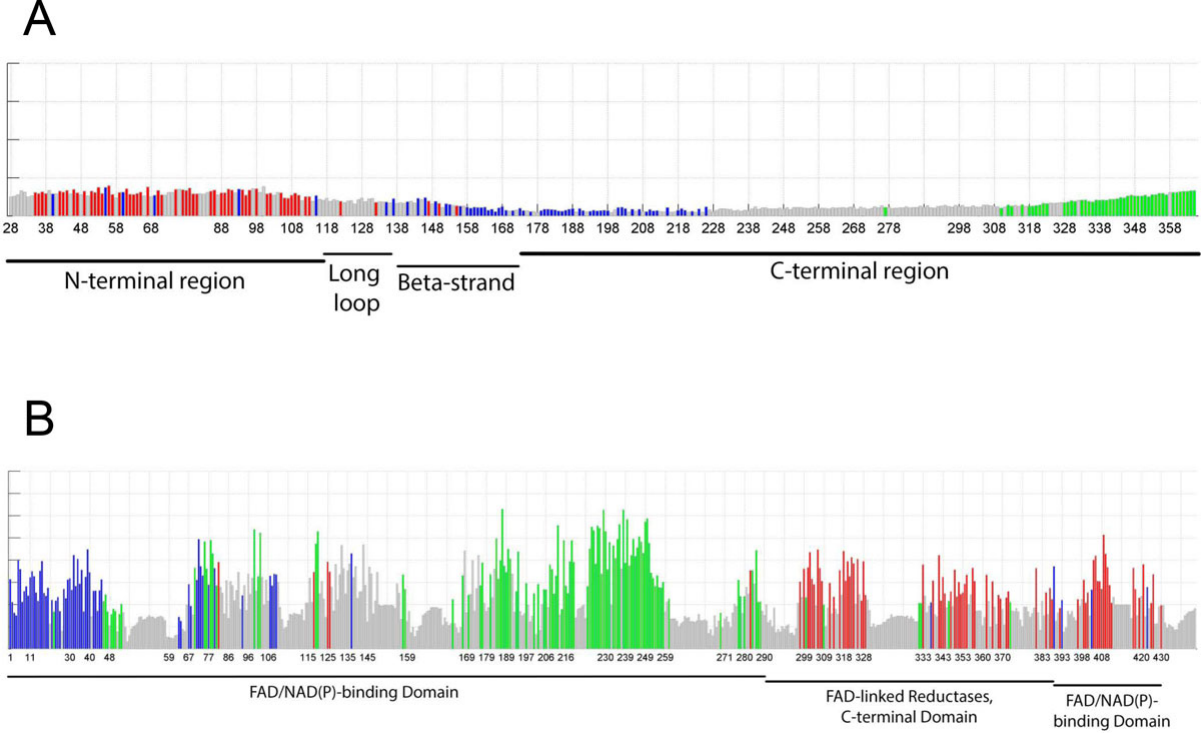
**Identification of protein sectors in the primary structure of SMS and GDI1**. A) Distribution of the protein sectors within the different regions of human spermine synthase. The analyzed residues correspond from position 28 to 365 based on the structure (PDB: 3C6K). The pattern observed appears to correspond to a stratification of sectors where the N-terminal region associated with dimerization appears to dominate the red sector, whereas the C-terminal region, ranging from residues 173 to 366, is composed of the blue and green sectors. B) Distribution of the protein sectors in Rab GDP dissociation inhibitor. The analyzed residues correspond from positions 1 to 447 based on the PDB structure file (1LV0). The N-terminal FAD/NAD(P) binding domain appears to be predominantly composed of residues belonging to the green and blue sectors. The FAD-linked reductase and the C-terminal FAD/NAD(P) binding domains appear to have a majority of residues classified within the red sector with a small presence of amino acids belonging to the blue and green sectors.

**Figure 3:**
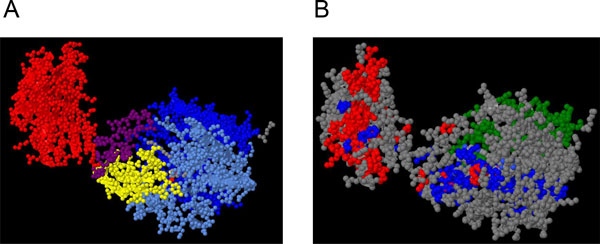
**Representation of protein domains and identified sectors in the 3D structure of spermine synthase (PDB: **3C6K**)**. A) Protein domains were colored in one of the chains of the homodimer. Red corresponds to the N-terminal domain, the long loop is in purple, the beta strand domain is in yellow, and the C-terminal region is in blue, with the catalytic center shown in light blue. B) Identified protein sectors in spermine synthase. Each sector is represented with a different color, and appears to have a distribution that is not limited to the described domains.

The protein sector analysis of both SMS and GDI1 revealed the distribution of groups of co-evolving residues (Figure 2a and 2b) that present a degree of spatial separation which differs from that found in protein domains (Figure 3a and 3b). When analyzing the sectors of SMS, we were able to identify three groups classified as the red, blue and green sector. Our results indicated that the red and blue sectors appear to be in the N-terminal region and to some extent the long loop and the beta strand domain (Figure 2a). However, the presence of residues of the red sector appears to be limited to these three regions, whereas the blue and green sectors span the C-terminal region, with no red sector residues identified in this region.

For GDI1, our analysis identified three distinct sectors, red, blue and green. The FAD/NAD(P) binding domain, located in the N-terminal region of GDI1, had amino acid residues classified within the blue and green sector (Figure 2b), with only three positions (82, 125 and 283) identified as belonging to the red sector. When analyzing the FAD-linked reductase and the FAD/NAD(P) binding domain located in the central and C-terminal regions of the protein, the residues in both are mostly classified as being part of the red sector (Figure 2b), with reduced presence of residues classified into the green and blue sectors. Although sector distribution of residues appears to follow a pattern where one group of residues is predominant over the others in a specific region, our analysis revealed that even within these regions, residues belonging to different sectors were identified (Figure 2a and 2b). This finding supports a degree of differentiation between protein sectors and domains.

Our results indicate that although protein sectors may be distributed across the proteins, there is a concentration of these co-evolving residues in certain areas of the proteins (Figure 2a and 2b), with stretches of residues that were not identified to be part of any sector. This observation could be attributed to the presence of conserved catalytic centers or active sites, necessary for protein function. One example was residue 276 in SMS (Table 1), which is a known active site that was not assigned to any sector. For GDI1, residue 92 (Table 1) has been reported to be necessary for the binding and recycling of RAB3 [27], and mutations in this site could lead to a reduction in its activity. This residue was also not classified in any of the three identified sectors. Because such important residues tend to be highly conserved, it is possible that these sites are not prone to undergo the co-evolutionary process.

**Table 1:** List of the residues associated with diseases in spermine synthase and Rab GDP dissociation inhibitor.

	Residue	Sector	Domain/Region	Effect/Associated Phenotype
**Spermine Synthase**	G56A	Red	N-terminal	Snyder-Robinson syndrome. Expanded phenotype of disease reported in [23]
	G67A	Red	N-terminal	New Snyder-Robinson syndrome phenotype. Expanded phenotype of disease reported in [30]
	V132A	Red	N-terminal/long loop	Snyder-Robinson syndrome. Expanded phenotype of disease reported in [24]
	I150A	Red	Central (beta-strand)	Decrease the stability of the C-terminal region, Snyder-Robinson syndrome
	Y328A	Green	C-terminal/catalytic	Mild mental retardation, Snyder-Robinson syndrome

**GDI1**	L92P	-	FAD/NAD(P) binding domain	Non-specific mental retardation
	R70TER	Green	FAD/NAD(P) binding domain	Non-specific mental retardation
	R423P	Blue	FAD/NAD(P) binding domain	Non-specific mental retardation

The disease-associated sites were obtained from OMIM and publications, and the list is organized according to the residue location in the protein domains.

### Disease-associated residues in protein sectors

The protein sector analysis of our test cases revealed a possible link between different disease phenotypes and the locations of the mutations within groups of co-evolving residues. We were able to identify multiple residues, in which disease-causing mutations have been identified (Table 1). As stated in Halabi et al. [6], groups of co-evolving residues seem to be associated with specific functions. In SMS, we identified the presence of a sector within the N-terminal region, which is necessary for protein dimerization. It has been reported that the ability to form dimers is necessary for full protein functionality. In our analysis, we found that the most severe phenotypes for the Snyder-Robinson Syndrome were predominantly found in the red sector, with residues mapped not only to the N-terminal region but also in the long loop and beta-strand domains (Figure 2a and 3a).

The results suggest that protein sectors, when compared to domains, allow for the clustering of mutations based on the underlying process of residue co-evolution instead of grouping them by sequential or spatial locations. The protein sector analysis of SMS has shown that the known Snyder-Robinson syndrome phenotypes, including expanded phenotypes [30], are mainly related to the red sector (Table 1), which comprises the N-terminal region as well as the long loop and beta strand domain. Thus, a protein sector can be associated with diverse functions as previously reported [6]. It appears that the red sector in SMS comprises regions associated with both dimerization and protein stability (Table 1), and these spatially distant regions may have undergone the co-evolutionary process because of their roles in the function of the protein. Interestingly, another mutation in SMS has been mapped to the green sector. This residue at position 328 (Table 1) has been associated with a milder form of the Snyder-Robinson syndrome [31]. It is possible that the green sector in the C-terminal region comprises residues associated with the enzyme's substrate recognition [28]. This may lead to a decrease in enzymatic activity, thus possibly accounting for the milder form of the syndrome [28].

The analysis of GDI1 identified two known mutations located in the green and blue sectors (Table 1). These amino acid positions (70 and 423) are associated with non-specific X-linked intellectual disability [26,27]. The two residues are located in spatially separated regions, with the residue at position 70 in the N-terminal FAD/NAD(P)-binding domain and the residue at position 423 in the C-terminal region (Figure 2b). It is still unknown whether these two mutations, distributed in different sectors, may cause any difference in disease phenotypes. Further studies are needed to explain our findings and expand the knowledge about this protein, including the possible role of the co-evolving resides in disease. The third residue at position 92 has been proposed as an important substrate binding site [26,27], and appears not to be undergoing co-evolution, probably owing to its significance in stabilizing the Rab-binding region or recognition of the C-terminal prenyl group in substrate recycling [27]. This may explain why our analysis did not identify it in any of the three sectors.

Our results from the protein sector analysis of SMS suggest a possible link between groups of co-evolving residues and disease severity. However, to fully understand the role of protein sectors, the use of mutagenic analysis can provide further information. Experimental validation of our results would present further evidence to support the approach of clustering disease-associated residues with their corresponding clinical manifestation. In addition, although we have presented a pipeline useful for clustering mutations, it is possible that other factors, such as the type of amino acid substitutions, could be the underlying cause of the differences in disease severity. Nevertheless, by clustering residues into different sectors, our approach can be used as a valuable aid in further characterizing the possible causes of disease such as intellectual disability. Protein sector analysis may also provide useful information for understanding the effect of mutations at spatially distant positions on disease phenotypes.

## Conclusions

We have proposed an integrative approach that makes use of the statistical coupling analysis method for the study of disease-causing mutations in proteins. Our test cases provided information regarding the role of protein sectors and how they could be associated to disease variants. We were also able to identify the effect of mutations in distinct sectors with variations in the clinical signs of a disease. These findings support the possible role of the protein sectors in specific functions that when affected could lead to variable phenotypes associated with a complex syndrome.

## Competing interests

The authors declare that they have no competing interests.

## Authors' contributions

LW conceived and oversaw the study. JG designed the study, conducted the data analysis, and drafted the manuscript. LW and CES contributed to result interpretation and manuscript preparation.

## Declarations

The publication of this work is funded by CSREES/USDA under project number SC-1000675.

This article has been published as part of *BMC Genomics *Volume 15 Supplement 11, 2014: Selected articles from the 2014 International Conference on Advances in Big Data Analytics. The full contents of the supplement are available online at http://www.biomedcentral.com/bmcgenomics/supplements/15/S11.
